# Measures of frailty in population-based studies: an overview

**DOI:** 10.1186/1471-2318-13-64

**Published:** 2013-06-21

**Authors:** Kim Bouillon, Mika Kivimaki, Mark Hamer, Severine Sabia, Eleonor I Fransson, Archana Singh-Manoux, Catharine R Gale, G David Batty

**Affiliations:** 1 Department of Epidemiology and Public Health, University College London, 1-19 Torrington Place, London WC1E 6BT, UK; 2 Finnish Institute of Occupational Health, Helsinki, Finland; 3 Institute of Environmental Medicine, Karolinska Institutet, Stockholm, Sweden; 4 School of Health Sciences, Jönköping University, Jönköping, Sweden; 5 INSERM U1018, Centre for Research in Epidemiology & Population Health, Villejuif F-94807, France; 6 Centre de Gérontologie, Hôpital Ste Périne, AP-HP, Paris, France; 7 MRC Lifecourse Epidemiology Unit, University of Southampton, Southampton, UK; 8 Centre for Cognitive Ageing and Cognitive Epidemiology, University of Edinburgh, Edinburgh, UK

**Keywords:** Frailty, Frail elderly, Measure, Overview, Reliability, Validity

## Abstract

**Background:**

Although research productivity in the field of frailty has risen exponentially in recent years, there remains a lack of consensus regarding the measurement of this syndrome. This overview offers three services: first, we provide a comprehensive catalogue of current frailty measures; second, we evaluate their reliability and validity; third, we report on their popularity of use.

**Methods:**

In order to identify relevant publications, we searched MEDLINE (from its inception in 1948 to May 2011); scrutinized the reference sections of the retrieved articles; and consulted our own files. An indicator of the frequency of use of each frailty instrument was based on the number of times it had been utilized by investigators other than the originators.

**Results:**

Of the initially retrieved 2,166 papers, 27 original articles described separate frailty scales. The number (range: 1 to 38) and type of items (range of domains: physical functioning, disability, disease, sensory impairment, cognition, nutrition, mood, and social support) included in the frailty instruments varied widely. Reliability and validity had been examined in only 26% (7/27) of the instruments. The predictive validity of these scales for mortality varied: for instance, hazard ratios/odds ratios (95% confidence interval) for mortality risk for frail relative to non-frail people ranged from 1.21 (0.78; 1.87) to 6.03 (3.00; 12.08) for the Phenotype of Frailty and 1.57 (1.41; 1.74) to 10.53 (7.06; 15.70) for the Frailty Index. Among the 150 papers which we found to have used at least one of the 27 frailty instruments, 69% (n = 104) reported on the Phenotype of Frailty, 12% (n = 18) on the Frailty Index, and 19% (n = 28) on one of the remaining 25 instruments.

**Conclusions:**

Although there are numerous frailty scales currently in use, reliability and validity have rarely been examined. The most evaluated and frequently used measure is the Phenotype of Frailty.

## Background

The global population of elderly people aged 60 years or more was 600 million in 2000; it is expected to rise to around 2 billion by 2050 [1]. With an aging population, researchers are increasingly interested in frailty [2,3], a syndrome characterized by age-related declines in functional reserves across an array of physiologic systems. Frail older adults experience an increased risk of a number of adverse health outcomes such as comorbidity, disability, dependency, institutionalization, falls, fractures, hospitalization, and mortality [4-21]. Identification of frail adults is important as trial evidence suggest that frailty status might be reversible with the implementation of exercise programs or hormone treatment [22-25].

A series of frailty measures have emerged in recent years. The aim of this overview is three-fold: 1) provide a comprehensive catalogue of existing frailty measures; 2) review evidence on the validity and reliability of these measures; and 3) quantify the popularity of each frailty measure by investigators other than the originators.

## Methods

### Search strategy

We took three approaches. First, we searched the electronic database MEDLINE (1948 to May 2011) through the OvidSP interface for all articles using the keyword “frailty” (using the term “frail” yielded an unmanageably large literature with little relevance to the present aims). This strategy allowed us to identify articles where this keyword appeared at least once in the title, abstract, or subject heading. Second, the reference sections of the retrieved articles were scrutinized for additional relevant papers by manual searches. Third, we searched our own records which included interrogation of our own relational databases (e.g. Reference Manager, Endnote). This overview followed the guidelines for the Meta-analysis of Observational Studies in Epidemiology (MOOSE) [26].

### Selection criteria

We included studies with participants aged 50 years and older at baseline examination in which the authors purport to have measured frailty. Further inclusion criteria were: 1) articles written in English, French, or Spanish; and 2) articles describing the reliability and validity of a frailty instrument.

### Assessment of the reliability and validity of frailty measures

The reliability and validity were assessed using suggested guidelines [27,28]. Reliability, which determines if a scale measures an entity (here frailty) in a reproducible way, was investigated through the following definitions: internal consistency (the average of the correlations among all items in the measure), intra-rater reliability (the agreement between observations made by the same rater on two different occasions), inter-rater reliability (the agreement between different raters), and test-retest reliability (the agreement between observations on the participants on two occasions separated by an interval of time). Validity – whether the scale in question measures what it purports to – was assessed by criterion and construct validity. Criterion validity refers to how well the instrument predicts an outcome. When frailty and the outcome data are collected simultaneously, the criterion validity is referred to as the concurrent validity. When the outcome data are prospectively collected, it is called predictive validity. Finally, in this context, construct validity refers to the extent to which a frailty measure correlates with factors that are, based on the extant literature, known to have an association (e.g. age, comorbidity, disability, physical capabilities or performances) [27,28].

### Use of frailty measurements by researchers

To evaluate the level of utilization of a given frailty instrument by researchers, we counted, among the selected articles, the number of publications which had been authored by researchers other than the originators in the periods ≤ 2000, 2001-2005, and ≥2006. In addition to this, we used the Scopus citation database [29] of peer-reviewed literature to analyze the number of citations in original research articles, excluding those cited by the creators of a given frailty instrument, for each frailty scales up to October 2011. In order to have an indication about the level of predictive validity of the identified frailty instruments, estimates – hazard ratios (or relative risks) and odds ratios – for the association between a frailty score and an adverse health outcome, in particular mortality, were examined.

## Results

**Figure 1:**
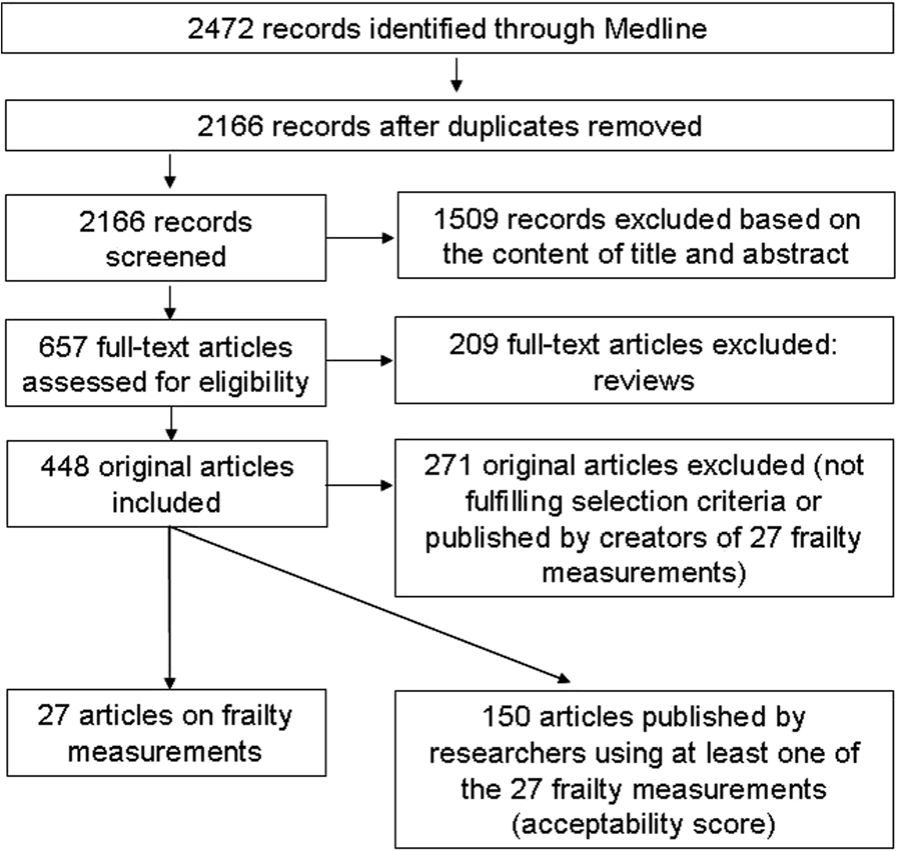
Phases of the literature search.

The initial keyword search using “frailty” identified 2,166 articles (Figure 1). Based on the content of the title and the abstract, 1,509 articles were excluded for the following reasons: article not published in English, French, or Spanish; article untraceable; studied population not of interest (animals, non-elderly population); statistical methods paper; or topic of the articles was not focused on measurement of frailty but its mechanism, predictors, prevention, intervention, and management/treatment. A further 209 papers were excluded because they were reviews rather than empirical papers. Of the remaining 448 articles, 27 [30-56] described the construction or psychometric properties of measures of frailty, and were included in this review. Among them, five instruments initially created to assess disability [57], vulnerability [58], and physical capabilities or performances [59-61] were used subsequently to assess frailty [36,39,41,42,44]. For these five instruments, their reliability, validity, and use were studied as a measure of frailty. A further 150 articles either applying or testing the validity of these 27 frailty measurements were included in our synthesis.

### Classifications: self-report, objective, and mixed frailty measures

All 27 identified frailty measurements were grouped into three categories (Additional file 1: Table S1): subjective (self-reported items only), objective (inclusion of only directly measured components), or subjective and objective combined (mixed). Eleven of the 27 instruments included only subjective components which were either reported by a participant (self-evaluation) in nine out of 11 cases [30-34,36,38-40], or reported by a clinician or a researcher (hetero-evaluation) [35,37]. Of the 27 frailty instruments, five included only objective components [41-45]. Finally, the remaining 11 instruments included both subjective and objective (mixed) components [46-56].

### General description of frailty measurements

Of the 27 frailty assessments, 19 were developed in population-based samples [30-32,34-37,40-44,46-48,50,51,53,55], 7 among hospitalized patients [33,39,45,49,52,54,56], and 1 without specification [38]. Half of the frailty scales (n=14) were created by research groups in the USA [30, 31,36,39,41-44,46-49,53,56], five in Canada [32,34,37,52,54], three in the Netherlands [33,40,51], two in Italy [38,45], and one each in Australia [55], France [50], and Sweden [35]. Five of the 27 frailty instruments were adapted from those developed initially to assess functional status [57], vulnerability [58], or physical performances [59-61]. These were used to measure frailty for the first time by Cacciatore and colleagues [36], Kanauchi and colleagues [39], Brown and colleagues [41], Gill and colleagues [42], and Bandinelli and colleagues [44], respectively. Furthermore, recently tested tools assessing frailty such as Static/Dynamic Frailty Index [51], Study of Osteoporotic Fractures Index [53], FRAIL scale [55], and Comprehensive Assessment of Frailty [56] were based on the Fried’s frailty scale [47] and/or the Mitnitski’s Frailty Index [34].

All identified frailty measures were composed of at least two items, except that of Gerdhem and colleagues [35] where a general assessment of health is made within a 15-second observation by the investigator. Of the subjective and mixed frailty measures, most contained disability and/or comorbidity components. Instruments without disability or comorbidity information were: the 1994 Frailty Measure [31], Subjective Frailty Score [35], Tilburg Frailty Indicator [40] all objective measures (Modified Physical Performance Test [41], Physical Frailty Score [42], Klein’s frailty index [43], Short Physical Performance Battery [44], and Opasich’s frailty scale [45]), Speechley & Tinetti’s frailty scale [46], Fried’s frailty scale [47], Score-Risk Correspondence for dependency [50], Study of Osteoporotic Fractures Index [53], and Brief Frailty Index [54]. Further descriptions of characteristics of population and type of components included in each instrument are also provided in (Additional file 1: Table S1).

### Assessment of the reliability and validity of frailty measures

Additional file 2: Table S2 presents reliability and validity data taken from the original articles and other related articles on the frailty measurements. Three approaches were used for reliability assessment: internal consistency, inter-rater, and test-retest reliability. Concurrent and predictive validity were mainly assessed using outcomes such as mortality, institutionalization, activities of daily living (ADL) disability, hospitalization, and quality of life. Only 7 out of 27 instruments (26%) were found to have had both reliability and validity ascertained [33,35,37,40,43,49,52].

Of all, 19 instruments had either their reliability or validity assessed. Among them, 4 instruments were tested for validity only once in the original sample/cohort of participants [32,36,55,56], and the Phenotype of Frailty by Fried and colleagues [47] and the Frailty Index by Mitnitski and colleagues [34] had their concurrent or predictive validity assessed in more than 3 samples/cohorts (17 and 13 samples/cohorts, respectively). One instrument out of 27, the Short Physical Performance Battery, previously used to assess physical functioning [61], had neither reliability nor validity information in measuring frailty [44].

Information on the predictive validity was available for 16 instruments. In 69% (n=11/16), the predictive validity was quantified by relating the frailty measure to mortality. With average follow-ups varying from 1 month to 12 years, hazard ratios or relative risks (from Cox regression) or odds ratios (from logistic regression) for mortality risk for frail people relative to those with no record of the condition ranged from 1.21 (95% confidence interval (CI): 0.78; 1.87) to 6.03 (95% CI: 3.00; 12.08) for the Phenotype of Frailty [47] and 1.57 (95% CI: 1.41; 1.74) to 10.53 (95% CI: 7.06; 15.70) for the Frailty Index [34]. The Phenotype of Frailty has been rarely used in a continuous fashion. One exception is Kulminski et al who found an increased mortality risk of 2% (RR=1.02; 95% CI: 1.02; 1.03) for a one unit of increase in this scale. For the Frailty Index, the estimates ranged from 1.008 (95% CI: 1.005; 1.011) to 10.53 (95% CI: 7.06; 15.70). The estimates – hazard ratios (or relative risks) and odds ratios – examining the association between a frailty score and mortality do not allow to affirm which score is the best in the prediction of mortality for several reasons: 1) relative risks and odds ratios are calculated differently [62]; 2) estimates were assessed in different populations, therefore with different baseline risks; 3) follow-ups and adjustment for confounding factors were heterogeneous. In spite of these limits, the estimates in Additional file 2: Table S2 give a qualitative appreciation on the magnitude of the association between a frailty score and mortality.

### Use of frailty instruments

**Figure 2:**
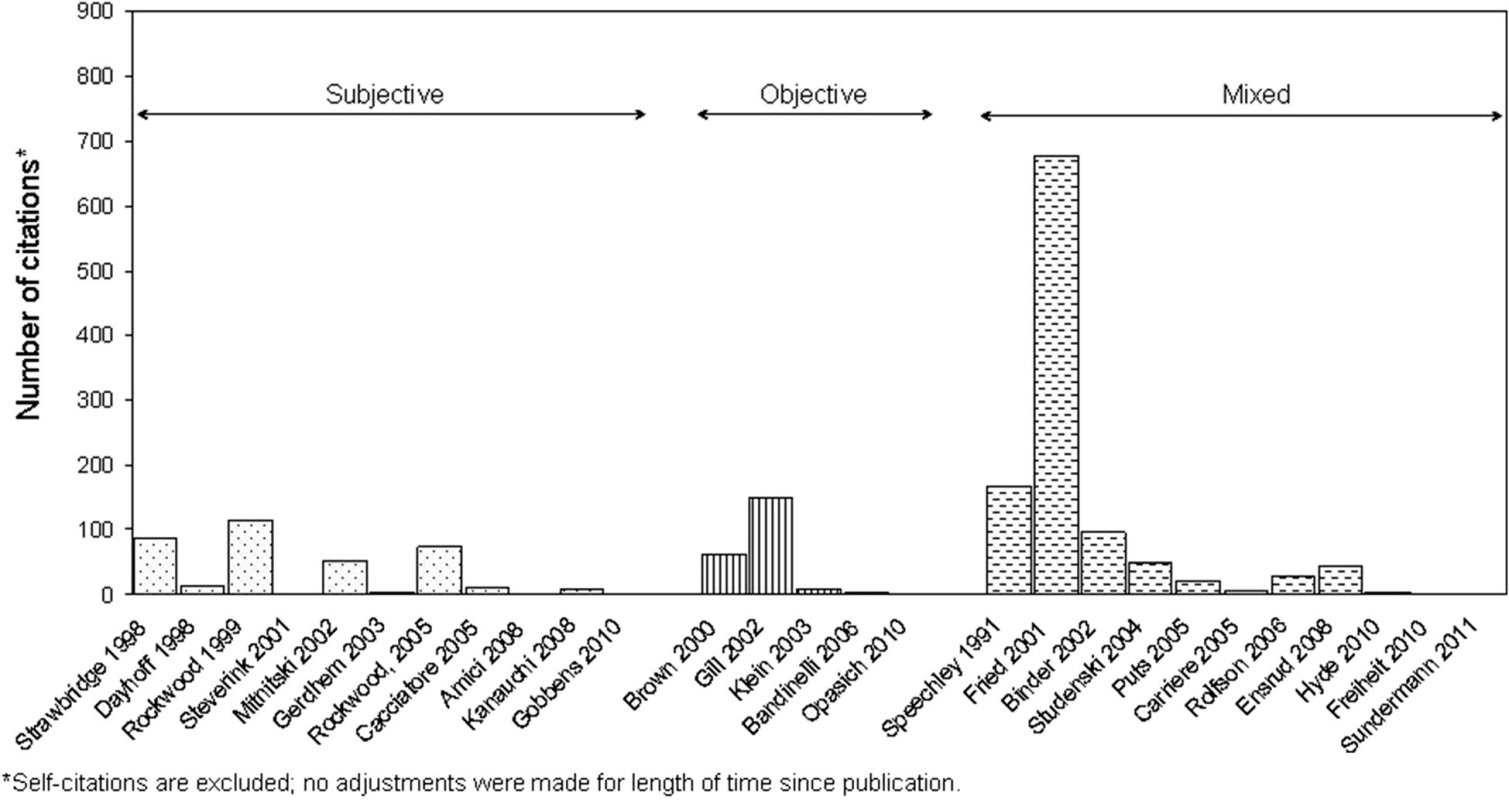
Number of original research articles citing individual frailty instruments according to the Scopus Citation Database, October 2011.

Additional file 3: Table S3 presents the number of publications in which a frailty measure had been used by investigators other than those who created it. In 69% of publications, a frailty scale developed by Fried and colleagues [47] was utilized; 12% used the Frailty Index developed by Mitnitski and colleagues [34]; 4% the Edmonton Frail Scale [52]; and ≤ 2% used the remaining instruments. This analysis also shows that half the frailty instruments (n=14) have not been employed at all by other researchers [30,35,36,38,43-45,48-51,54-56]. Figure 2 displays the number of original research articles based on the Scopus citation database, which referenced one of the 27 frailty instruments: the 3 most cited papers were that of Fried and colleagues, 2001 [47] (n=676), Speechley and colleagues, 1991 [46] (n=167), and Gill and colleagues, 2002 [42] (n=150). The citation rank for Mitnitski and colleagues’ paper, 2002 [34] was ninth (n=52).

## Discussion

In this overview, we aimed at providing a comprehensive catalogue of frailty measures, reviewing evidence on their validity and reliability, and quantifying the use of each measure by investigators other than the originators. We identified 27 frailty scales used in 150 studies to date. We made a series of observations. First, although frailty, disability, and comorbidity are inter-related, they are distinct clinical entities [63,64]. Integrating disability or comorbidity items into a frailty scale may be debatable as they are not equivalent concepts. However, half the frailty instruments (n=14) include either disability or comorbidity components [30,32-34,36-39,48,49,51,52,55],[56]. Second, at least five measures [36,39,41,42,44] of frailty were originally created to measure vulnerability, functional status, and physical performances, suggesting a lack of terminological rigor. Third, we observed that four recent scales [51,53,55,56] are based on existing measures, in particular the Fried scale. Finally, confusion between frailty scales can be generated because sometimes a specific instrument is named differently in different studies (the Fried scale [47] being labelled as Fried Frailty Index [65] on occasion). Elsewhere, several instruments are identically named but have different item content: for instance, the term “frailty index” was used by different researchers [34,43,54]. This was also the case with “frail scale” [52,66].

### Assessment of the reliability and validity of frailty measures

The Standards for Educational and Psychological Testing [67], a guideline which describes the best practice in the development of complex measures such as frailty, recommends the reporting of the basic principles of test construction such as reliability and validity. However, this information was available only for a few instruments: CSHA Clinical Frailty Scale [32] and Edmonton Frail Scale [52]. They had acceptable reliability (Kappa coefficient ≥ 0.7) and good concurrent and predictive validity. Two instruments were widely tested for their validity but not reliability: the Frailty Index [34] and the Fried’s scale [47]. Reliability and validity are the most important indicators when selecting one measure over another. However, even among 7 frailty measurements with such information [33,35,37,40,43,49,52], none of them appear to be recognized as a “gold standard”. Comparing the performances of different frailty scales in predicting an objective health outcome such as mortality was complicated by the use of different confounding factors across studies.

In several studies, investigators have examined the inter-relationships between different measures of frailty. Thus, the Fried’s scale has been compared with the Frailty Index [10,68,69] and the Study of Osteoporotic Fracture index [15,53] using different methods: correlation analyses [69], comparison of strength of cross-sectional [68] and prospective associations [10,15], and use of the c-index statistic [53]. The Fried’s scale is moderately well correlated with the Frailty Index [69], and shows a stronger association with age and sex (important criteria of construct validity [28]) [68] but a weaker association with mortality [10]. The Fried’s scale and the Study of Osteoporotic Fracture index have a similar strength of association with falls, disability, hospitalization [15] and death [53]. As Streiner and Norman [27] highlighted, we found that it was sometimes difficult to disentangle whether an assessment belongs to concurrent validity or construct validity. Therefore, certain classifications in either category might be arguable.

### Use of the frailty instruments

We attempted to assess the use of a frailty instrument by counting the number of publications that had adopted the instrument other than the original creators. The two instruments which have had their external validity most extensively evaluated against adverse health outcomes were those developed by Fried group (Phenotype of Frailty) and Mitnitski group (Frailty Index). These are based on two different conceptual frameworks. The Fried group has suggested that frailty represents a phenotype which reflects underlying age-related changes in multiple systems. By contrast, the Mitniski group advances that frailty is the accumulation of multiple deficits, with the degree of frailty denoted by the number of such deficits. This highlights that although some investigators recognize that frailty, comorbidity, and disability are distinct entities [28,47,70], for others they are overlapping. Most reviews or editorials on frailty have implicitly presented the Phenotype of Frailty as standard [63,71-81] whereas for others the standard is the Frailty Index [82,83]. Recommendations from other researchers are more nuanced. For Sternberg and colleagues [84], the choice depends on the definition and outcomes that best suit the investigators or clinicians responsible for the screening. The European, Canadian and American Geriatric Advisory Panel [66] recommend using a hybrid measure, the “FRAIL” scale, comprising components from both the Phenotype of Frailty and the Frailty Index.

The Fried’s scale [47] has been the most extensively tested for its validity and is the most widely used instrument in frailty research [65,78,85-134]. Randomized controlled trials have also used the scale to screen elderly participants [24,25,135-140], or as an outcome of interventions [22,23,139]. The Fried’s scale is widely used, allowing comparisons to be made between studies.

The main limitation of our assessment of use of these instruments is that it penalizes the more recently published frailty instruments. However, the Fried’s scale is not the oldest measure in the field and several more recent frailty instruments are either derived or similar to that measure, suggesting that qualities other than duration of availability explain the popularity of this instrument. Another limitation lies in the lack of elimination of articles that may have resulted from the original authors’ circle of influence. For example, some of the articles which report on the use of the Fried’s scale may have been produced from former co-workers who had previously utilized the CHS data – the dataset in which the Fried’s scale was first validated.

In spite of its wide use, the Fried’s scale has some drawbacks common to other frailty instruments. Chiefly, different scales utilize different classification of the individual components. For example, in the Cardiovascular Health Study (CHS), participants were considered positive for weight loss if they reported having lost more than 10 pounds unintentionally in the last year or they objectively lost 5% or more in comparison with the previous year’s body weight [47]. In Women’s Health Aging Study-I, however, a cut-off of 10% in comparison with the self-reported weight at age 60 years [4] was utilized. These important variations in the operationalization of frailty measurement render comparisons of findings between studies as problematic.

In addition to the manual counting procedure to estimate the use of the frailty instruments, we also examined the number of citations in original research articles (excluding those cited by the creators of a given frailty instrument) for the 27 papers describing the frailty instruments. Even though the rank of citations was different for some of the frailty instruments than that of the manual counting, the paper on the Fried’s scale was still the most highly cited. Although the number of citations can be easily accessed, this electronic database search cannot replace the manual counting method as the papers citing the original articles do not necessarily use the tool in question.

Among previously published reviews [66,83,84,141-145] on frailty measures, only one [83] assessed them in terms of reliability and validity. Compared with the De Vries and colleagues’ paper [83], our review presents additional strengths. First, to evaluate reliability and validity of a given instrument, we have extracted data from other studies, reflecting its level of external validation. Second, to our knowledge, no article has been published on the extent to which frailty measures have been used by other researchers. This finding might reflect the level preference of researchers for a given frailty measurement in the absence of a consensually recognized tool. Moreover, we identified 18 other frailty instruments [30,32,35-38,40-46,48,52,54-56], 5 of them created in 2010 and after. Another limitation of our review may lie in the use of a unique keyword “frailty” to identify relevant publications on frailty measurements. One may find such a strategy restrictive, leading to miss some screening tools helping to identify frail elderly. In fact, we included similar frailty instruments than those comprised in the recent reviews [83,84].

## Conclusions

This review provides a comprehensive overview of existing frailty measurements. We identified 27 measures of frailty but none of them have been recognized as a gold standard. Difficulties in accepting one measure as a reference may lie in the following reasons: the existence of frailty as a clinical entity is quite new; the definition of frailty is still debatable, therefore, it is difficult to create a composite measure that would meet all criteria. Components to include in the frailty instruments need to be further discussed to reach a consensus, in particular on whether to include disability and/or disease data. The most widely used frailty measurements by investigators [34,47], such as the frailty scales developed by Fried and colleagues and Mitnitski and colleagues need to be further assessed, including attempts to improve them, before being recognized as a gold standard.

## Abbreviations

ADL: Activities of daily living; CHS: Cardiovascular health study; CSHA: Canadian study of health and aging.

## Competing interests

The authors declared that they have no competing interests.

## Authors’ contributions

All authors contributed to writing this review and have approved the final version for publication.

## Pre-publication history

The pre-publication history for this paper can be accessed here:

http://www.biomedcentral.com/1471-2318/13/64/prepub

## Supplementary Material

Additional file 1: Table S1 Characteristics of frailty instruments utilized in individual studies [30-54,146]. Click here for file

Additional file 2: Table S2 Reliability and validity results for frailty instruments utilized in individual studies [147-173]. Click here for file

Additional file 3: Table S3 Use of subjective, objective and mixed frailty instruments by type and publication year [68,147,148,174-239]. Click here for file
