# Supplementary material for: Measures of frailty in population-based studies: an overview
Source: BMC Geriatr. 2013 Jun 21;13:64. doi: 10.1186/1471-2318-13-64 (PMC3710231; doi:10.1186/1471-2318-13-64)
Supplement: Additional file 1: Table S1 — Characteristics of frailty instruments utilized in individual studies [30-54,146]. [file 1471-2318-13-64-S1.doc]

Table S1. Characteristics of frailty instruments utilized in individual studies

| **Reference/*Frailty instrument name*** | **Study name, setting, country** | **Characteristics of population:**  **N, age (mean (SD); range),**  **% female** | **Components** | **Classification** | **Comment** |
| --- | --- | --- | --- | --- | --- |
| **Subjective frailty instruments** | | | | | |
| Strawbridge et al, 1998 [31]: *1994 Frailty Measure* | The Alameda County Study,  Prospective cohort,  USA | Community-dwelling population  N=574  74.0 years; 65+  57.0% | 4 domains:  Physical functioning:   - Sudden loss of balance - Weakness in arms - Weakness in legs - Dizziness when standing up quickly   Nutritive functioning:   - Loss of appetite - Unexplained weight loss   Cognitive functioning:   - Difficulty paying attention - Trouble finding the right word - Difficulty remembering things - Forgetting where put something   Sensory problems:   - Difficulty reading a newspaper - Difficulty in recognizing a friend across the street - Difficulty reading signs at night - Hearing over the phone - Hearing a normal conversation - Hearing a conversation in a noisy room | Score for the 6 sensory items:  1: have no difficulty  2: have a little difficulty  3: have some difficulty  4: have a great deal of difficulty.  Scores on the other 10 items:  1: rarely or never had the problem in the last 12 months  2: sometimes had the problem  3: often had the problem  4: very often had the problem  Participant was considered to have a problem or difficulty for one domain when he/she had a score ≥3 at least 1 of the items.  Frail if ≥ 2 domains were considered to have a problem or difficulty. |  |
| Dayhoff et al, 1998 [30] | Subsample of a larger study examining effects of two exercise interventions,  Cross-sectional analysis,  USA | Community-dwelling participants  N=84  Non-frail: 73.2 years (6.0)  Frail: 73.5 years (7.9)  Age range : 60 to 88 years  85.7% | - Performance of ADLs/IADLs using the World Health Organisation Assessment of Functional Capacity (14 items, each scored from 1 to 5 (5=unable to perform)) - Self-report of perceived health. | Score range:  14 (self-sufficiency) to 70 (total dependency)  Non-frail if score ≤20 & excellent/good health.  Frail if score ≥21 & fair/poor health | Frailty defined as disability. |
| Rockwood et al, 1999 [32]:  *CSHA rules based definition* | The Canadian Study of Health and Aging (CSHA),  Prospective cohort,  Canada | Random sample of community residents  N=not reported  65+  %=not available | - 0: Those who walk without help, perform basic ADL, are continent of bowel and bladder, and are not cognitively impaired - 1: Bladder incontinence only - 2: One (two if incontinent) or more of needing assistance with mobility or ADL, has cognitive impairment with no dementia, or has bowel or bladder incontinence - 3: Two (or three if incontinent) or more of totally dependent for transfers or one or more ADL, incontinent of bowel and bladder, and diagnosis of dementia. | -- | Frailty defined as disability or comorbidity. |
| Steverink et al, 2001 [33]:  *Groningen frailty indicator (GFI)*  (manual search) | Cross-sectional study,  Netherlands | Hospital inpatients, nursing home residents and community-dwelling elderly  N=275  78.0 years (7.0), range=64-99  72.9% | 15 items scored 0 or 1:   - Mobility (4 items) - Comorbidity - Malnutrition - Cognition - Vision - Hearing - Physical energy - Loneliness (3 items) - Depressed mood - Anxiety feelings | Frail if score ≥ 5 out of 15. | Frailty defined as disability or comorbidity.  Need further explanation in the GFI construction. |
| Mitnitski et al, 2002 [34]:  *Frailty index (FI)* | The Canadian Study of Health and Aging (CSHA),  Prospective cohort,  Canada | Random sample of community residents  N=2914  82.0 years (7.4); 65+  64.4% | 20 “deficits” (symptoms, signs, impairments and disabilities) | Impairment index: 0 to 1 | No clear cut-off between frail vs non-frail.  No standardised number and type of deficits.  Frailty defined as disability or comorbidity. |
| Gerdhem et al, 2003 [35]:  *Subjective Frailty Score* | Cross-sectional analysis  Sweden | Participants randomly selected from the city files of Malmo  N=993  75 years  100% | To make a general assessment of health and appearance within 15 sec from first sight, and transfer this into an arbitrary scale. | Score ranging from 1 (low frailty) to 100 (very frail). | No clear cut-off between frail vs non-frail. |
| Rockwood et al, 2005 [37]:  *Canadian Study of Health and Aging Clinical Frailty Scale (CSHA-CFS)* | The Canadian Study of Health and Aging (CSHA),  Prospective cohort,  Canada | Random sample of community residents  N=2305 | 7-point:  1: Very fit  2: Well  3: Well, with treated comorbid disease  4: Apparently vulnerable  5: Mildly frail  6: Moderately frail  7: Severely frail (complete functional dependence on others) | Moderately frail: 6  Severely frail: 7 | Frailty defined as disability or comorbidity.  Needs a clinical interview. |
| Cacciatore et al, 2005 [36]:  *Frailty Staging System*  Based from Lachs et al, 1990, USA [57] | Osservatorio Geriatrico Regione Campania,  Prospective cohort,  Italy | Random sample of subjects with/without chronic heart failure, community-dwelling or institutionalised elderly  N=1332  75.9 years (6.7)  60% | 7 core domains of functioning scored 0 (function is preserved) or 1 (function is lost):   - BADL disability - Mobility (ability to do heavy housework, to walk up and down stairs to the second floor and to walk half a mile) - Cognitive function - Visual function - Hearing function - Urinary continence - Social support | Class 1: 0 or 1  Class 2: 2 or 3  Class 3: ≥4 | Frailty defined as disability. |
| Amici et al, 2008 [38]: *Marigliano-Cacciafesta Polypathological Scale (MCPS)* | Cross-sectional design,  Italy | N=180  79.5 years; 70+  63.9% | - Neurological disorders (5 items) - Cardiopathy (4 items) - Respiratory disorders (5 items) - Renal disorders (4 items) - Locomotive apparatus disorders (5 items) - Sensory deprivation (5 items) - Metabolism and nutritional state (5 items) - Cognitive state and mood (5 items) - Peripheral vascular system ( 5 items) - Malignant cancerous disorders (5 items) - Gastroenteritic disorders (5 items) | Score range: 0 to 245.  Polypathology:  Slight: <15  Medium: 15-24  Medium-severe: 25-49  Severe: 50-74  Very severe: 75+ | Missing information about population characteristics  Rationale for weighting scores not explained.  Frailty defined as comorbidity.  Dose-response effect not shown. |
| Kanauchi et al, 2008 [39]:  Based on Morris et al, 1984, USA [146]:  *Hebrew Rehabilitation Center for Aged (HRCA) Vunerability Index* and Saliba et al, 2001, USA [58]: *Vulnerable Elders Survey-13 (VES-13)* | Cross-sectional study,  Japan | Hospital inpatients with cardiometabolic risk factors  N=101  72.9 years (5.1); range 65-85  43.6% | *HRCA Vulnerability Index* (2 components):  A component includes self-reported requirements for help in:   - Preparing meals (score 0 or 1) - Taking out the garbage (score 0 or 1) - Doing ordinary work around the house (score 0 or 1) - Walking up and down stairs (score 0 or 1) - Needing to use a cane (score 0 or 1) - Needing to use a walker (score 0 or 1) - Identifying the current year (score 0 or 1)   B component includes self-reported answers for:   - Leaving their residence (score 0 or 1) - Needing help in dressing (score 0 or 1) - Having health impediments (score 0 or 1)   *VES-13* (13 items):   - Age (score 0 to 3; 3 if ≥85) - Self-reported health (score 0 or 1) - Difficulties in physical activities (6 items) (score 0 to 2) - ADLs/IADLs (5 items) (score 0 or 4) | *HRCA Vulnerability Index*::  Vulnerable if A component score>1 or A component score=1 and B component>0  *VES-13* :  Score range: 0 to 10  Frail if score >3  Participants were frail if they were considered as vulnerable according to the *HRCA Vulnerability Index* or frail according to the *VES-13* | Frailty defined as disability. |
| Gobbens et al, 2010 [40]:  *Tilburg Frailty Indicator (TFI)* | Cross-sectional design,  Netherlands | 2 random samples of community-dwelling participants  Sample 1: n=245, 80.3 years (3.9), 54.7%  Sample 2: n=234, 80.2 years (3.7), 59.0% | 15 items scored 0 or 1:  8 physical domains:   - Feeling physically healthy - Unexpected weight loss - Difficulty in walking - Difficulty in maintaining balance - Hearing problems - Vision problems - Lack of strength in hands - Physical tiredness   4 psychological domains:   - Cognition - Depressive symptoms - Anxiety - Coping   3 social domains:   - Living alone - Social relations - Social support | Score range: 0 to 15 (15=highest score for frailty) | No clear cut-off between frail vs non-frail. |
| **Objective frailty instruments** | | | | | |
| Brown et al, 2000 [41]: *Modified Physical Performance Test (PPT)*  Based on Reuben & Siu, 1990, USA [59]: *PPT* and Guralnik et al, 1995, USA [61] | Cross-sectional analysis,  USA | Community-dwelling elderly  N=107  83 years (4); 77+  %=not available | 9 items scored 0 to 4:   - Lift a 7-pound book to a shelf - Put on and remove a jacket - Pick up penny from floor - Performance of a 360 degrees turn - 50-foot walk test - Climb one flight of stairs - Climb up and down 4 flights of stairs - Stand up 5 times from a 16-inch chair - Progressive Romberg test | Score range: 0-36  Not frail: 32-36  Mild frailty: 25-32  Moderate frailty: 17-24  Dependent: <17 |  |
| Gill et al, 2002 [42]  Based on Gill et al, 1995, USA [60] | Primary care practices,  Randomized controlled trial,  USA | Community-dwelling elderly  N=188  Intervention group:  n=94, 82.8 years (5.0); 75+, 80%  Control group:  n=94, 83.5 years (5.2); 75+, 70% | - Rapid gait (walking back and forth over a 10-foot (3-m) course as quickly as possible) - Single chair stand | Moderately frail if rapid gait>10 s or could not stand from the chair.  Severely frail if meet both criteria. |  |
| Klein et al, 2003 [43]:  *Frailty index* | Beaver Dam Eye Study,  Prospective cohort,  USA | Sample from a private census of the population of Beaver Dam  43+ years | - Timed 10-ft walk (score=1 if in the highest quartile, stratified by sex) - Handgrip strength (score=1 if in the lowest quartile, stratified by sex) - Peak expiratory flow rate (score=1 if in the lowest quartile, stratified by sex) - Ability to stand from a sitting position without using arms in one try (score=1 if unable) | Score range: 0 (better) to 4 (worse) |  |
| Bandinelli, 2006 [44]:  *Short Physical Performance Battery (SPPB)*  Based on Guralnik et al, 1995, USA [61] | The FRAilty Screening and Intervention trial,  Italy | Community-dwelling elderly visiting their primary care physicians  N=251  Treatment group:  n=126, 76.4 years (3.6), 66%  Control group:  n=125, 76.4 years (3.4), 60% | 3 items scored 0 (unable to perform complete the test) to 4 (highest level of performance):   - Walking speed over 4 metres - 5 timed repeated chair rises - Standing balance | Score range: 0 to 12  Frail if ≤9 |  |
| Opasich et al, 2010 [45] | Hospital based, study of effect of personalized versus usual physiotherapy, Italy | Patients after receiving a cardiac surgery procedure  N=224  Intervention group:  n=150, 74.6 years (3.6); 70+, 40%  Control group:  n=74, 75 years (3.9); 70+, 45% | - Balance Performance Oriented Mobility Assessment (BPOMA): assessment of static and dynamic balance - Get-Up-and-Go (GUG) test | Non-frail:  BPOMA>19 and GUG ≤10s  Moderately frail: BPOMA≤19 or GUG >10s  Severely frail: BPOMA≤19 and GUG >10s |  |
| **Mixed (subjective and objective) frailty instruments** | | | | | |
| Speechley & Tinetti, 1991 [46] | Subsample of the Yale Health and Aging Project (YHAP) of the Established Populations for Epidemiologic Study of the Elderly (EPESE) program  Prospective cohort,  USA | Community dwelling elderly  N=336  75+ years | Frail attributes (each item scored 0 or 1):   - Age ≥80 years - Gait/balance abnormalities - Infrequent walking for exercise - Depressed - Taking sedatives - Decreased strength in shoulder - Decreased strength in knee - Lower extremity disability - Near vision loss   Vigorous attributes (each item scored 0 or 1):   - Age <80 years - Cognitively intact - Frequent physical exercise other than walking - Good near vision | Score:  0-9 frail attributes  0-4 vigorous attributes  Frail: ≤1 vigorous and ≥4 frail attributes.  Vigorous: ≥3 vigorous and ≤2 frail attributes.  Transitional: neither frail nor vigorous. |  |
| Fried et al, 2001 [47]:  *Phenotype of Frailty* | Cardiovascular Health Study (CHS),  Prospective cohort,  USA | Community dwelling elderly from 4 US communities  N=5317  65+ years  57.9% | 5 items, each scored 0 or 1:   - Unintentional weight loss - Self-reported exhaustion - Weakness (grip strength) (1 if in the lowest quintile) - Slow walking speed (1 if in the highest quintile) - Low physical activity (1 if in the lowest quintile) | Score range: 0 to 5  0: frail  1-2: pre-frail  ≥3: frail |  |
| Binder et al, 2002 [48]:  *Physical frailty* | Randomized controlled trial,  USA | Community-dwelling elderly  N=444  83 years (4); 78+  65.8% | - Modified Physical Performance Test score (see Brown et al, 2000) of 18-32 - Peak oxygen consumption: 11-18 ml/kg - Self-reported difficulty or need for assistance in 2 instrumental ADL or 1 basic ADL | Mild to moderate frailty if ≥2 | Instrument contained disability component.  Instrument used exclusively to select mild to moderate frailty elderly in randomized controlled trials. |
| Studenski et al, 2004 [49]:  *Clinical Global Impression of Change in Physical Frailty (CGIC-PF)* | Qualitative and quantitative instrument development,  USA | N=not available  80.7 years (6.4)  80% | - Appearance (3 indicators) - Healthcare utilisation (3 indicators); - Medical complexity (3 indicators) - Strength (3 objective measures) - Balance (3 self-reported+objective measures) - Nutrition (3 objective measures) - Stamina (2 indicators) - Neuromotor (3 objective measures) - Mobility (4 objective measures) - Perceived health (1 indicator) - ADL (4 indicators) - Emotional status (2 indicators) - Social status (4 indicators) | Change evaluated after 6 months of follow-up, scored from 1 (worse) to 7 (better). | Needs a clinical interview.  No clear cut-off between frail vs non-frail.  Frailty defined as disability / comorbidity. |
| Puts et al, 2005 [51]:  *Static/Dynamic frailty index* | Longitudinal Aging Study Amsterdam (LASA),  Prospective cohort,  Netherlands | Random sample drawn from registers  N=1152  Range: 55-85 years  52.3 to 60.0% | - Body mass index - Peak expiratory flow - Cognition - Vision and hearing problems (self-reported) - Incontinence (self-reported) - Sense of mastery (Pearlin & Schooler Mastery scale) - Depressive symptoms (CES-D) - Physical activity | Static frail if ≥3 components.  Dynamic frail if decline or loss ≥3. | Inclusion of one item of disability.  Inspired from Fried et al’s instrument. |
| Carriere et al, 2005 [50]:  *Score-Risk Correspondence for dependency* | Epidemiologie de l’Osteoporose (EPIDOS) study,  Prospective cohort,  France | Random sample drawn from vote-registration or health-insurance membership rolls  N=545  Median age (interquartile range): 79 years (76-81); 75+  100% | - Time (years) since baseline evaluation - Age (>=74 years) X Time since baseline evaluation - Mobility - Gait speed<0.78 m/s - Time (s) to complete 5 chair stands - Perceived health - Fear of falling - Time (s) to stand in tandem position - Body mass index - Grip strength - Physical activity - Education | Score: 25-169  Risk: 0.02-0.99 | No clear cut-off between frail vs non-frail. |
| Rolfson et al, 2006 [52]:  *Edmonton Frail Scale (EFS)*  (manual research) | Hospital based,  Cross-sectional analysis,  Canada | Sample of patients referred for a comprehensive geriatric assessment (CGA)  N=158  80.4 years (6.8); 65+  53% | - Cognition (drawing a clock) (score 0 to 2) - General health status (2 questions each scored 0 to 2) - Functional independence (score 0 to 2) - Social support (score 0 to 2) - Medication use (2 questions each scored 0 to 1) - Nutrition (score 0 to 1) - Mood (score 0 to 1) - Continence (score 0 to 1) - Functional performance (score 0 to 2) | Score 0-17 (17=highest level of frailty) | No clear cut-off between frail vs non-frail.  Frailty defined as disability. |
| Ensrud et al, 2008 [53]:  *Study of Osteoporotic Fractures (SOF) index* | Study of Osteoporotic Fractures,  Prospective cohort,  USA | Community-dwelling elderly from population-based listings in 4 areas of USA  N=6701  76.7 years (4.8); 69+  100% | 3 items each scored 0 to 1:   - Unintentional weight loss (≥5% in 2 years) - Inability to rise from a chair 5 times without using arms - Reduced energy level (Geriatric Depression Scale) | Robust: 0  Pre-frail:1  Frail: >=2 | Inspired from Fried et al’s instrument. |
| Hyde et al, 2010 [55]:  *FRAIL scale* | Health in Men Study,  Prospective cohort,  Australia | Random sample of community-dwelling elderly from the electoral roll  N=3616  76.9 years (3.6); 71+  0% | 5 items each scored 0 to 1:   - Fatigue (SF-36) - Resistance - ability to climb a single flight of stairs (SF-36) - Ambulation - ability to walk one block (SF-36) - Illnesses - more than 5 (list of 14 diseases) - Loss of weight - more than 5% (between 4 to 5 years) | Frail if ≥3 | Frailty defined as comorbidity.  Inspired from Fried et al’s and Mitnitski’s instruments. |
| Freiheit et al, 2010 [54]:  *Brief Frailty Index* | Substudy of the Calgary Cardiac and Cognition (3C) Study  Prospective cohort study, hospital-based,  Canada | Patients with coronary artery disease  337  70.8 years (5.9); 60+  27% | 5 items each scored 0 to 1:   - Balance assessment - Body mass index - Trail-Making Test Part B - Geriatric Depression Scale - Living alone | Index score range: 0-5 (high score=high risk)  4 categories:  0; 1; 2; ≥3 |  |
| Sundermann et al, 2011 [56]: *Comprehensive Assessment of Frailty (CAF)* | Hospital-based,  Prospective study,  USA | Patients undergoing cardiac surgery  N=400  80.1 years (4.0); 74+  51.5% | Modified Fried et al’s phenotype of frailty criteria, each scored 0 or 1:   - BMI score - Exhaustion score - Physical activity score - Slowness score (walking 4 mm in usual gait speed) - Weakness score (grip strength)   Physical performance tests, each scored 0 to 4:   - Standing static Balance - Chair rise - Put on and remove a jacket - Pick up a pen from floor - Turn 360 degrees   Laboratory tests, each scored 0 to 1:   - Serum albumin score - Forced expiratory volume in 1 second - Creatinine score   Rockwood et al’s CSHA-CFS scored 1 to 7 | Score range: 1-35  Not frail: 1-10  Moderately frail: 11-25  Severely frail: 25+ | Based on Fried et al’s and Rockwood et al’s instruments. |

“Manual search” characterizes an article not referenced by Medline but found in the reference section of selected articles.
