# Supplementary material for: Measures of frailty in population-based studies: an overview
Source: BMC Geriatr. 2013 Jun 21;13:64. doi: 10.1186/1471-2318-13-64 (PMC3710231; doi:10.1186/1471-2318-13-64)
Supplement: Additional file 2: Table S2 — Reliability and validity results for frailty instruments utilized in individual studies [147-173]. [file 1471-2318-13-64-S2.doc]

**Table 1. Reliability and validity results for frailty instruments utilized in individual studies**

| **Frailty instrument** | **Population** | **Reliability** | **Validity** | **Strength of the association between frailty measure and mortality (estimate with its 95% CI)^d^** |
| --- | --- | --- | --- | --- |
|  |  | **Type: statistical analysis** | **Type: outcome/statistical analysis** |  |
| **Subjective** | | | | |
| Strawbridge et al, 1998 [31]:  *1994 Frailty Measure* | The Alameda County Study [31]; sample of outpatients from a geriatric practice [147]; the Health Retirement Study [68] | None | **Concurrent validity**: quality of life [31]; cognitive impairment, ADL & IADL [68]/logistic regression  **Construct validity**: physical performance measures [147]/Pearson’s correlation coefficient | NA |
| Dayhoff et al, 1998 [30] | Not reported [30] | None | **Construct validity**: balance test & muscle strength [30]/discriminant analysis | NA |
| Rockwood et al, 1999 [32]:  *CSHA rules based definition* | The CSHA [32] | None | **Predictive validity**: mortality and institutionalisation [32]/Cox’s proportional hazards modelling | **Rockwood** [32]: FU=5 y, RR=3.1 (2.7; 3.6)**^a^** |
| Steverink et al, 2001 [33]:  *Groningen frailty indicator* | Hospital inpatients, nursing home residents, and community-dwelling elderly [33]; sample of community dwelling elderly [148] | **Internal consistency**: Cronbach’s alpha=0.76 [33]; 0.73 [148] | **Concurrent validity**: MOS SF20 & GHQ [33]/t-test; disability (GARS)/Spearman’s rank correlation coefficient  **Internal construct validity**: principal component analysis [33]  **Construct validity**: TFI & SPQ [148]/Spearman’s rank correlation coefficient | NA |
| Mitnitski et al, 2002 [34]:  *Frailty index* | The CSHA [34,37,69]; the Cardiovascular Health Study [10]; the Health Retirement Study [68]; a Chinese health survey [149]; the US National Long Term Care Survey [150]; the US Medicare Current Beneficiary Survey [151]; the Chinese longitudinal healthy longevity survey [152,153]; the Mexican Health and Aging Study [154]; home care clients of 8 community Care Access Centres [155]; 7 population-based and 4 clinical/institutional surveys in 4 developed countries [156]; the Gothenburg H-70 cohort study [157]; the Conselice Study of Brain Ageing [158]; the National Population Health Survey of Canada [159] | None | **Predictive validity**: mortality [10,34,37,69,149-151,154-156,158,159], hospitalization [151], institutionalisation [151,155]/Cox’s proportional hazards modelling; mortality [152]/multinomial logistic regression; mortality [153]/Weibull hazard regression; mortality , institutionalisation [157]/Kaplan-Meier method  **Concurrent validity**: cognitive impairment, ADL & IADL [68]/logistic regression  **Construct validity**: age [34,149] | **Kulminski** [10]: FU range=4 y, RR=1.05 (1.04; 1.06)**^b^**  **Mitnitski** [34]: median FU (death)=2.8 y, RR=1.008 (1.005; 1.011)^b^  **Rockwood** [37]: FU range=5.8 y, HR=1.26 (1.24; 1.29)^b^  **Rockwood** [69]: HR and its CI not reported.  **Goggins** [149]: FU range=10 y, RR=1.28 (1.23; 1.33)^c^  **Hastings** [151]: FU range=30 d, RR=1.98 (1.29; 3.05)^a^  **Garcia-Gonzalez** [154]: FU range=2 y, HR=6.45 (4.10; 10.14)^a^  **Armstrong** [155]: FU range=1 y, HR=1.93 (1.79; 2.08)^a^  **Mitnitski** [156]: FU range=12 y, HR=1.03 (1.03; 1.04)^b^  **Lucicesare** [158]: FU range=4 y, HR=5.26 (1.05; 26.42)^b^  **Song** [159]: FU range=10 y, RR=1.57 (1.41; 1.74)^a^  **Dupre** [152]: FU range=over 3 y; RRR(men)=7.75 (5.54; 10.83)^a^; RRR(women)=10.53 (7.06; 15.70)^a^  **Gu** [153]: FU range=3 y; RR(men)=4.56 (2.68; 6.44)^a^; RR(women)=3.84 (1.86; 5.72)^a^ |
| Gerdhem et al, 2003 [35]:  *Subjective Frailty Score* | Sample of participants living in Malmo, Sweden [35] | **Inter-rater reliability**: Spearman rank correlation=0.51 to 0.59 [35] | **Construct validity**: gait, balance, muscle strength, fall [35]/Spearman rank correlation | NA |
| Rockwood et al, 2005 [37]:  *CSHA Clinical Frailty Scale* | The CSHA [37]; sample of geriatric outpatients [160] | **Inter-rater reliability**: intraclass correlation coefficient=0.97 [37]; weighted kappa=0.68 [160] | **Predictive validity**: mortality [37], institutionalisation [37]/Cox’s proportional hazards modelling  **Construct validity**: modified MMSE, Cumulative Illness Rating Scale, history of falls, delirium, cognitive impairment or dementia, CSHA rules-based definition of frailty, CSHA Frailty Index, CSHA Function Scale [37]/Pearson or Spearman correlation coefficient; physician version & Phenotype of Frailty [160]/weighted kappa & Kendall’s tau correlation | **Rockwood** [37]: FU range=5.8 y, HR=1.30 (1.27; 1.33)^b^ |
| Cacciatore et al, 2005 [36]  *Frailty Staging System* | Osservatorio Geriatrico Regione Campana [36] | None | **Predictive validity**: mortality [36]/Cox’s proportional hazards modelling | **Cacciatore** [36]: FU range=12 y, HR=1.62 (1.08; 2.45)^a^; HR=1.48 (1.04; 2.11)^b^ |
| Amici et al, 2008 [38]:  *Marigliano-Cacciafesta Polypathological Scale* | Sample of patients [38] | None | **Concurrent validity**: mini nutritional assessment, Tinetti test, Barthel index, global evaluation functional index, geriatric depression scale [38]/ Pearson’s correlation coefficient | NA |
| Kanauchi et al, 2008 [39]  *Vulnerable Elderly Survey-13* | Patients in nephrology [39]; geriatric outpatients [161]; the Medicare Current Beneficiary Survey [162] | None | **Predictive validity**: mortality [161], fracture [161], cancer diagnosis [162]/logistic regression  **Concurrent validity**: WHO quality of life [39]/multi-way ANCOVA | **Ma** [161]: FU range=6 y, OR=1.16 (0.98; 1.37)^b^ |
| Gobbens et al, 2010 [40]:  *Tilburg Frailty Indicator* | Samples of community dwelling elderly [40,148] | **Internal consistency**: Cronbach’s alpha=0.73 [40]; 0.79 [148]  **Test-retest reliability**: Pearson correlation coefficient=0.79 [40] | **Predictive validity**: disability [40], health care utilisation [40]/linear regression & ROC analyses  **Concurrent validity**: disability (GARS) [148]/Spearman’s rank correlation coefficient; WHO quality of life [40]/multiple regression analyses  **Construct validity**: GFI & SPQ [148]/Spearman’s rank correlation coefficient; 15 single TFI components [40]/Pearson’s correlation | NA |
| **Objective** |  |  |  |  |
| Brown et al, 2000 [41]:  *Modified Physical Performance Test* | Community-dwelling elderly [41] | None | **Construct validity**: obstacle course, Romberg full tandem, Berg balance test, fast gait [41]/ANOVA | NA |
| Gill et al, 2002 [42]:  *Physical Frailty Score* | Participants living in the municipality of Treviso [163]; the Precipitating Events Project longitudinal study [159,164] | None | **Predictive validity**: mortality [163], ADL [159,164]/Cox’s proportional hazards modelling  **Concurrent validity**: ADL & IADL [163]/Chi-square test | **Gallucci** [163]: HR and its CI not reported. |
| Klein et al, 2003 [43]:  *Frailty index* | Sample from a private census of the population of Beaver Dam [43] | **Inter-item consistency**: Spearman and Pearson correlation coefficients=0.31 to 0.52 [43] | **Concurrent validity**: distance visual acuity and contrast sensitivity [43] | NA |
| Bandinelli, 2006 [44]:  *Short Physical Performance Battery* | Patients recruited by primary care physicians [44] | None | None | NA |
| Opasich et al, 2010 [45] | Medically stable patients after a cardiac surgery procedure [45] | None | **Concurrent validity**: functional impairment, disability, post-surgery course [45]/2-factor analysis of variance | NA |
| **Mixed** |  |  |  |  |
| Speechley & Tinetti, 1991 [46] | The Yale Health and Aging Project cohort [46] | None | **Predictive validity**: falls [46]/Chi-2 test for trend in proportion  **Internal construct validity**: principal component analysis [46] | NA |
| Fried et al, 2001 [47]:  *Phenotype of Frailty* | The Cardiovascular Health Study [10,47,165]; the MacArthur Study [11]; the Health Retirement Study [68]; Toufen, Taiwan [166]; Sample of women [53]; the Maintenance of Balance, Independent Living, Intellect, and Zest in the Elderly Boston Study [15]; the Osteoporotic Fractures in Men study [7]; the Study of Osteoporotic Fractures [8]; the Three-City Study [9]; the Hispanic Established Population for the Epidemiological Study of the Elderly [12,14,167,168]; the Concord Health and Ageing in Men Project [18]; the Montreal Unmet Needs Study [20]; the Women’s Health and Aging Studies I & II [6]; the Women’s Health Initiative Observational Study [5]; a nationwide Survey of Health and Living Status of the Elderly in Taiwan [169]; the Canadian Study of Health and Aging [69]; sample of surgical patients [170] | None | **Predictive validity**: mortality [5-10,14,47,53,69,168], fractures [5,8,53], falls [15,47], ADL & IADL [6,12,47], hospitalisation [47], institutionalisation [6,69], idiopathic venous thromboembolism [165]/ Cox’s proportional hazards modelling; mortality [11], falls [8,53], ADL & IADL [5,9,47,53], hospitalization [5,9,15], emergency department visits [15]/logistic regression; MMSE [171]/ general linear mixed model; postoperative complications [170]/logistic regression model  **Concurrent validity**: ADL & IADL [15,68]; Bartel index score & depression [166], use of specific health and community services [18]/logistic regression; chronic medical conditions [15], SPPB [15], MMSE [15], Hopkins Verbal Learning Test [15]; Trail Making Test part A & part B [15], Clock-in-a-Box [15], CESD scale [15]/analyse of variance; ADL & IADL, comorbidity [20]/the Cochran-Mantel-Haenszel test; ADL & IADL, comorbidities [169]/one-way ANOVA; health-related quality of life using SF-36 [167]/logistic regression model  **Internal construct validity**: latent class analysis [6]  **Convergent validity**: Mitnitski’s Frailty Index score [69]/Pearson’s correlation coefficient | **Woods** [5]: mean FU=5.9 y, HR=1.71 (1.48; 1.97)**^a^**  **Bandeen-Roche** [6]: FU range =3 y, HR=6.03 (3.00; 12.08)**^a^**  **Cawthon** [7]: mean FU=4.7 y, HR=2.05 (1.55; 2.72)**^a^**  **Ensrud** [8]: mean FU=9 y, HR=1.82 (1.56; 2.13)**^a^**  **Avila-Funes** [9]: FU range=4 y, HR=1.21 (0.78; 1.87)**^a^**  **Kulminski** [10]: FU range=4 y, RR=1.02 (1.02; 1.03)**^b^**  **Sarkisian** [11]: FU range=9 y, OR=2.1 (1.2; 3.8)**^a^**  **Graham** [14]: FU range=10 y, HR=1.81 (1.41; 2.31)**^a^**  **Fried** [47]: FU range=7 y, HR=1.63 (1.27; 2.08)**^a^**  **Ensrud** [53]: FU range=9 y, HR=2.75 (2.46; 3.07)**^a^**  **Rockwood** [69]: HR and its CI not reported.  **Berges** [168]: FU range=10 y, HR(men)=3.04 (2.16; 4.28)**^a^**; HR(women)=1.92 (1.39; 2.65)**^a^** |
| Binder et al, 2002 [48]:  *Physical frailty* | Community-dwelling elderly [48] | **Test-retest reliability** for modified physical performance test=0.96 [48] | None | NA |
| Studenski et al, 2004 [49]:  *Clinical Global Impression of Change in Physical Frailty* | Sample of 24 patients [49] | **Inter-rater reliability**: Kendall’s multiple-rater concordance coefficient=0.97 [49] | **Face validity**: 6 experts & 46 clinicians [49] | NA |
| Puts et al, 2005 [51]:  *Static/Dynamic frailty index* | The Longitudinal Aging Study Amsterdam [51] | None | **Predictive validity**: performance tests (walking speed, rising from a chair, putting on and taking off a cardigan, and maintaining balance in a tandem stand) & ADL [51]/logistic regression | NA |
| Carriere et al, 2005 [50]:  *Score-Risk Correspondence for dependency* | The EPIDOS study [50] | None | **Predictive validity**: IADL [50]/logistic regression | NA |
| Rolfson et al, 2006 [52]:  *Edmonton Frail Scale* | Sample of patients 65+ years [52]; home care clients of 8 community Care Access Centres [155]; Toufen, Taiwan [166]; Brazilian elderly [172] | **Internal consistency**: Crohnbach’s coefficient=0.62 [52]  **Inter-rater reliability**: Kappa coefficient=0.77 [52] | **Predictive validity**: mortality [155], institutionalization [155]/Cox’s proportional hazards model; postoperative complications/logistic regression model  **Concurrent validity**: comorbidity [166], MMSE [166], incontinence [166], depression [166]/logistic regression  **Construct validity**: Barthel Index [52], Rolfson and colleagues’ GCIF [52]/Pearson correlation; MMSE score & the Functional independence measure [172]/Spearman’s correlation coefficient | **Armstrong** [155]: FU range=1 y, HR=2.49 (2.32; 2.68)^a^ |
| Ensrud et al, 2008 [53]:  *Study of Osteoporotic Fractures index* | Sample of women [53]; the Maintenance of Balance, Independent Living, Intellect, and Zest in the Elderly Boston Study [15]; community-dwelling outpatients [173] | None | **Predictive validity**: mortality [53], fractures [53], falls [15]/Cox’s proportional hazards; falls [53], disability [53], overnight hospitalization [15], emergency department visits [15]/logistic regression;  **Concurrent validity**: ADL & IADL [15]/logistic regression; chronic medical conditions [15], SPPB [15], MMSE [15], Hopkins Verbal Learning Test [15]; Trail Making Test part A & part B [15], Clock-in-a-Box [15], CESD scale [15]/analyses of variance; Older People’s quality of life [173]/linear regression analysis | **Ensrud** [53]: FU range=9 y, HR=2.37 (2.14; 2.61)**^a^** |
| Hyde et al, 2010 [55] :  *FRAIL scale* | The Health in Men Study [55] | None | **Predictive validity**: mortality [55]/Cox’s proportional hazards model; ADL & IADL [55]/logistic regression model | **Hyde** [55]: FU range=7 y, HR=3.97 (2.89; 5.45)**^a^** |
| Freiheit et al, 2010 [54]:  *Brief Frailty Index* | Patients undergoing cardiac catheterization for coronary artery disease [54] | None | **Predictive validity**: ADL [54], health-related quality of life [54]/Poisson regression model | NA |
| Sundermann et al, 2011 [56]:  *Comprehensive Assessment of Frailty* | Patients undergoing elective cardiac surgery [56] | None | **Predictive validity**: mortality [56]/Armitage’s trend test for proportions  **Construct validity**: Society of Thoracic Surgeons score & European system for cardiac operative risk evaluation [56]/Spearman’s rank correlation | NA |

Abbreviations: (I)ADL: (instrumental)activity of daily living; CI: confidence interval; CSHA: Canadian Study of Health and Aging; FU: follow-up; GARS: Groningen activity restriction scale; GHQ: general health questionnaire; HR: hazard rate; RR: relative risk; MMSE: mini-mental state examination; MOS-SF20: medical outcomes study 20-item short-form; NA: not available; OR: odds ratio; RRR: relative risk ratio; SPQ: Sherbrooke postal questionnaire.

^a^ RR calculated for the highest versus lowest category of the frailty score.

^b^ RR calculated based on 1-unit increment in the frailty score.

^c^ RR calculated based on 10-year increment in the frailty score.

^d^ The estimates – RRs and ORs – do not allow to affirm which frailty instrument better predicts mortality; however, they give a qualitative appreciation on the magnitude of the association between a given instrument and mortality.
