# Supplementary material for: Measures of frailty in population-based studies: an overview
Source: BMC Geriatr. 2013 Jun 21;13:64. doi: 10.1186/1471-2318-13-64 (PMC3710231; doi:10.1186/1471-2318-13-64)
Supplement: Additional file 3: Table S3 — Use of subjective, objective and mixed frailty instruments by type and publication year [68,147,148,174-239]. [file 1471-2318-13-64-S3.doc]

Table 1 Use of subjective, objective and mixed frailty instruments by type and publication year

|  | **Number of publications (%)* by year** | | |  |  |
| --- | --- | --- | --- | --- | --- |
| **Frailty instrument** | **Before 2000** | **2001-2005** | **2006 or later** | **All** | **References** |
|  | **N = 0** | **N = 8** | **N = 142** | **N = 150** |  |
| **Subjective** | | | | | |
| Strawbridge et al., 1998 [31]: | 0 | 1 (12.5) | 1 (0.7) | **2 (1.3)** | [68,147] |
| 1994 Frailty Measure |  |  |  |  |  |
| Dayhoff et al., 1998 [30] | 0 | 0 | 0 | **0** | - |
| Rockwood et al., 1999 [32]: | 0 | 0 | 0 | **0** | - |
| CSHA rules based definition |  |  |  |  |  |
| Steverink et al., 2001 [33]: | NA | 0 | 3 (2.1) | **3 (2.0)** | [148,174,175] |
| Groningen Frailty Indicator |  |  |  |  |  |
| Mitnitski et al., 2002 [34]: | NA | 2 (25.0) | 16 (11.2) | **18 (12.0)** | [10,68,103,149-155,176-183] |
| Frailty index |  |  |  |  |  |
| Gerdhem et al., 2003 [35]: | NA | 0 | 0 | **0** | - |
| Subjective Frailty Score |  |  |  |  |  |
| Rockwood et al., 2005 [37]: | NA | 0 | 3 (2.1) | **3 (2.0)** | [160,166,184] |
| CSHA Clinical Frailty Scale |  |  |  |  |  |
| Cacciatore et al., 2005 [36]: | NA | 0 | 0 | **0** | - |
| Frailty Staging System |  |  |  |  |  |
| Amici et al., 2008 [38]: | NA | NA | 0 | **0** | - |
| MCPS |  |  |  |  |  |
| Kanauchi et al., 2008 [39]: | NA | NA | 3 (2.1) | **3 (2.0)** | [161,162,174] |
| Vulnerable Elderly Survey-13 |  |  |  |  |  |
| Gobbens et al., 2010 [40]: | NA | NA | 1 (0.7) | **1 (0.7)** | [148] |
| Tilburg Frailty Indicator |  |  |  |  |  |
| **Objective** |  |  |  |  |  |
| Brown et al., 2000 [41]: | NA | 1 (12.5) | 1 (0.7) | **2 (1.3)** | [185,186] |
| Modified Physical Performance Test |  |  |  |  |  |
| Gill et al., 2002 [42]: | NA | 0 | 2 (1.4) | **2 (1.3)** | [163,187] |
| Physical Frailty Score |  |  |  |  |  |
| Klein et al., 2003 [43]: | NA | 0 | 0 | **0** | - |
| Frailty index |  |  |  |  |  |
| Bandinelli, 2006 [44]: | NA | NA | 0 | **0** | - |
| Short Physical Performance Battery |  |  |  |  |  |
| Opasich et al., 2010 [45] | NA | NA | 0 | **0** | - |
| **Mixed** | | | | | |
| Speechley & Tinetti, 1991 [46] | 0 | 3 (37.5) | 0 | **3 (2.0)** | [188-190] |
| Fried et al., 2001 [47]: | NA | 1 (12.5) | 103 (72.5) | **104 (69.3)** | [7-9,12-20,22,24,25,53,65,68,86,89-93,98-100,102-105,109,110,113-115,119-123,126,127,131-140,166-169,171,191-236] |
| Phenotype of Frailty |  |  |  |  |  |
| Binder et al., 2002 [48]: | NA | 0 | 0 | **0** | - |
| Physical frailty |  |  |  |  |  |
| Studenski et al., 2004 [49]: | NA | 0 | 0 | **0** | - |
| CGIC-PF |  |  |  |  |  |
| Puts et al., 2005 [51]: | NA | 0 | 0 | **0** | - |
| Static/Dynamic frailty index |  |  |  |  |  |
| Carriere et al., 2005 [50]: | NA | 0 | 0 | **0** | - |
| Score-Risk Correspondence for dependency |  |  |  |  |  |
| Rolfson et al., 2006 [52]: | NA | NA | 6 (4.2) | **6 (4.0)** | [65,155,166,172,237,238] |
| Edmonton Frail Scale |  |  |  |  |  |
| Ensrud et al., 2008 [53]: | NA | NA | 3 (2.1) | **3 (2.0)** | [15,173,239] |
| Study of Osteoporotic Fractures index |  |  |  |  |  |
| Hyde et al., 2010 [55]: | NA | NA | 0 | **0** | - |
| FRAIL scale |  |  |  |  |  |
| Freiheit et al., 2010 [54]: | NA | NA | 0 | **0** | - |
| Brief Frailty Index |  |  |  |  |  |
| Sundermann et al., 2011 [56]: | NA | NA | 0 | **0** | - |
| Comprehensive Assessment of Frailty |  |  |  |  |  |

Abbreviations: *CSHA*, Canadian study of health and aging; *MCPS*, Marigliano-cacciafesta polypathological scale; *CGIC-PF*, Clinical global impression of change in physical frailty.

*Number of publications/total number of publications during the period x 100.
